# Adverse effects of criminal legal system involvement: a qualitative study examining the role of incarceration and reentry on substance use trajectories among women with opioid use disorders

**DOI:** 10.1186/s44263-024-00058-1

**Published:** 2024-05-01

**Authors:** Sienna Strong-Jones, Kristina Brant, Derek Kreager, Eric Harrison, Abenaa Jones

**Affiliations:** 1 Department of Human Development and Family Studies, Pennsylvania State University, State College, PA 16801 USA; 2 Department of Agricultural Economics, Sociology, and Education, Pennsylvania State University, State College, PA 16801 USA; 3 Department of Sociology and Criminology, Pennsylvania State University, State College, PA 16801 USA

**Keywords:** Women, Criminal justice system, Criminal legal system, Substance use

## Abstract

**Background:**

Women with substance use disorders (SUDs) often experience adversity related to incarceration and reentry that can impact their substance use outcomes. This study aims to examine the adverse effects of incarceration and reentry on substance use outcomes among women with a history of opioid use disorder (OUD).

**Methods:**

We carried out 42 semi-structured interviews (May–July 2022) with women with a history of criminal legal involvement and OUD (*n* = 20), criminal legal professionals (*n* = 10), and SUD treatment professionals (*n* = 12). The interviews focused on women’s substance use trajectories, barriers to treatment, and the intersection of the criminal legal system and treatment. None of the women were presently incarcerated during their interviews.

**Results:**

Participants expressed the severe adverse impact of criminal legal involvement on women with OUD. Many women felt traumatized by experiencing detox while incarcerated, especially as they received minimal aid for withdrawal symptoms. Women seeking recovery while incarcerated felt unsupported, being afforded few treatment options, and experiencing stigma. Reentering society after incarceration also posed significant challenges to women’s individual recovery goals. Reentry-related challenges such as returning to unsupportive environments, facing difficulties finding employment, lacking secure housing, and facing the stigma of incarceration triggered adverse outcomes, such as relapse among those who were seeking to avoid illicit substances.

**Conclusions:**

Findings suggest a need to evaluate detox protocols, medication for opioid use disorder (MOUD) access, and stigma in the criminal legal system. Interventions facilitating women’s reentry, reducing the combined stigma of incarceration and OUD, and restoring agency for women with OUD are also needed.

**Supplementary Information:**

The online version contains supplementary material available at 10.1186/s44263-024-00058-1.

## Background

Opioids are the leading factor responsible for both fatal and non-fatal cases of overdose in the USA [1]. In 2021, a toll of over 80,000 Americans, including 23,652 women, died of an opioid-related overdose [2]. People with opioid use disorder (OUD) are at particular risk of facing both fatal and non-fatal overdose. As outlined in the Diagnostic and Statistical Manual of Mental Disorders, 5th Edition (DSM-V) [3], OUD is a chronic condition characterized by frequent opioid use, higher tolerance for opioids, withdrawal symptoms, unsuccessful cessation attempts, and impaired functioning, with biological, behavioral, and social factors contributing to its development [1, 4].

The primary purpose of an opioid prescription is pain management, and women who experience higher rates of acute and chronic pain have been prescribed opioids at higher rates compared to men; consequently, the prescribed use of opioids emerged as the primary avenue for women to develop OUD [5]. It is important to note that many women develop OUD via non-prescribed opioid use as well [6]; women may initially use prescription opioids that were not prescribed for them and transition to illicit opioids due to other risk factors, including peers that engage in illicit substance use [7], psychiatric disorders [8], child maltreatment [9], and victimization during adulthood [10]. Women with OUD frequently have a history of childhood maltreatment, especially sexual abuse (41%) [6]. Women who have undergone both early trauma and trauma across the life course may resort to opioid use as a means of self-medication or coping with adverse emotional conditions [11], heightening their susceptibility to OUD and risk of overdose [12]. The number of deaths caused by drug overdoses involving any opioid among women rose significantly, increasing from 2.6 per 100,000 population in 1999 to almost 16 (15.5) in 2017, marking a surge of 492% [13]. Yet, despite these increases, there has been limited awareness and acknowledgment of the extent of women’s exposure to opioids and the resulting consequences [5].

With an incarcerated population surpassing 2 million individuals [14], the USA has the highest rate of incarceration among high-income nations [15], and substance use histories are pervasive among incarcerated individuals [16]. Prisons, overseen by individual states and the federal government, are correctional facilities housing people who typically serve sentences exceeding 2 years for felony convictions [17]. In contrast, jails are under the jurisdiction of city or county authorities and usually hold individuals who are incarcerated pre-trial or are serving sentences of under 2 years [17]. Based on data from the 2007 and 2008–2009 National Inmate Surveys (NIS), a majority of individuals in state prisons (58%) and jails (63%) met the criteria for drug dependence or abuse according to the Diagnostic and Statistical Manual of Mental Disorders, 4th Edition (DSM-IV) and, in contrast, only around 5% of the general population aged 18 or older met these criteria [18].

Predominantly influenced by the criminalization of substance use [19, 20], the USA has also recently experienced a surge in the number of women incarcerated [21], with an 11% increase from 2008 to 2019 [22]. Women’s incarceration rate has not only been rising at a faster pace than men’s but there is also a disproportionate number of these cases linked to drug use [19], especially among pregnant individuals and mothers [23]. Roughly 7 out of 10 incarcerated women fulfill the DSM-IV criteria for drug dependence or abuse [24]. Many incarcerated women are of childbearing age, and approximately 3–4% are pregnant at intake [21]. Recent studies estimate that 14% of pregnant women who are incarcerated have an OUD diagnosis [23].

Despite the high prevalence of substance use among the incarcerated population, only a small portion of criminal legal agencies screen for OUD [25]. One recent study that reviewed the intake forms of a sample of U.S. jails found that 27% did not inquire about substance use at all [26]. An even smaller percentage of these agencies offer access to medications for opioid use disorder (MOUD), such as methadone, naltrexone, and buprenorphine [25, 27, 28]. Therefore, many individuals who were using MOUD before incarceration are unable to continue treatment while incarcerated and experience MOUD withdrawal when they enter jail [25]. This lack of provision of MOUD and SUD treatment generally is viewed as a breach of established norms [29], prompting individuals to engage in illicit drug transactions within correctional facilities to evade withdrawal symptoms [25].

While pregnant individuals tend to have greater access to MOUD, with about 60% of jails offering MOUD continuation for pregnant people, only about 34% of jails offer MOUD initiation during pregnancy [30, 31]. A separate study of 53 county jails found that approximately 50% of pregnant individuals with OUD endure withdrawal symptoms without any relief [27]. Moreover, most facilities discontinue the administration of MOUD to incarcerated individuals after childbirth, reflecting a prioritization of fetal well-being over maternal health [23]. Therefore, there are still considerable barriers to treatment for all incarcerated women [23], which may lead to substandard care and potentially harmful experiences.

Women with OUD often encounter challenges such as inadequate medical care and face difficulties related to victimization and intimidation in correctional settings [32–34]. One study found that 20.3% of women experienced physical victimization while incarcerated, and 15.3% experienced sexual victimization [35]. The ongoing experience of victimization while incarcerated adds to the complex trauma histories that many women with OUD have prior to entering the criminal legal system [36] and may affect their mental and physical well-being [31, 34]. Individuals may resort to self-medication to manage psychological distress that results from victimization [37], which can lead to an increased reliance on illicit substances among women experiencing victimization in correctional settings. There is also evidence of long-term adverse impacts of incarceration [38, 39]. One systematic review found that experiencing potentially traumatic events (PTEs), including direct victimization, during incarceration is positively associated with the development of post-traumatic stress disorder (PTSD) [38].

Due to inadequate medical care and distress among incarcerated individuals, overdose rates are prevalent in jail and prison: between 2009 and 2019, there was a fivefold increase in substance use-related mortality rates in prisons and a threefold increase in jails [40]. Moreover, 15% of all deaths were attributed to drug and alcohol intoxication in 2019, marking an increase from 4% in 2000 [41].

Fatal overdoses often happen shortly after admission, with individuals who experienced a fatal overdose spending a median duration of just 1 day incarcerated [41].

Upon release from incarceration, individuals encounter additional challenges as they navigate the transition back to society [28, 42, 43]. Prior research has shown that a considerable number of post-release deaths in the USA can be attributed to drug overdose [43, 44]. Drug overdose is the predominant cause of death following incarceration due to various environmental, social, and biological factors, including lack of access to OUD treatment, social network disruptions, and reduced tolerance while incarcerated, as outlined in the Post-Release Opioid Overdose Risk Model [45]. During the first 2 weeks following release, the likelihood of death from an overdose is 12.7 times greater compared to the general population, with women experiencing an even higher elevated risk of mortality [46].

While existing studies have documented the benefits of providing MOUD in carceral settings, the challenges faced by incarcerated individuals with OUD have yet to be comprehensively examined. Due to the large proportion of women incarcerated for drug-related offenses, it is imperative to investigate and address the unique challenges that incarceration and reentry pose for women who use drugs. This study aims to better understand the adversity experienced by women during incarceration and reentry and explore how this distress influences their substance use patterns.

To do so, we triangulate perspectives from legally involved women with OUD, SUD treatment professionals, and criminal legal professionals. Additionally, we seek to highlight the gender-specific effects of incarceration and reentry, recognizing that women may face unique challenges. This research is crucial given the persistent criminalization of substance use, as understanding the impact of incarceration and reentry on substance use trajectories can inform the design of tailored interventions and comprehensive support mechanisms aimed at addressing these issues.

## Methods

This study draws on 42 in-depth interviews conducted in Pennsylvania (PA) between May and July 2022. The study involved three distinct groups of participants: (1) women previously or currently using any type of MOUD (such as methadone, buprenorphine, or naltrexone) who had a history of criminal legal involvement (*N* = 20), (2) SUD treatment professionals working in community settings with women presently or formerly involved in the criminal legal system (*N* = 12), and (3) criminal legal professionals working with women affected by opioid use (*N* = 10) [47, 48].

This study includes the perspectives of these three groups, triangulating multiple perspectives that can offer additional insights from diverse stakeholders within interconnected groups [49]. In our investigation, women with OUDs who are involved in the criminal legal system engage with professionals in both the criminal legal and SUD treatment domains. These professionals actively influence the substance use trajectories of the women whom they work with and can offer additional insights into the systems in which they work. Although the individual perspectives of the women impacted by OUD and criminal legal involvement are crucial, professionals working with them may add a broader perspective on the factors that may promote or hinder well-being based on their experiences with a diverse spectrum of women.

As this study is set in PA, it is important to mention those local- and state-specific policies that influence MOUD access in correctional facilities. PA jails operate independently within each county, leading to significant variation in the availability of MOUD across facilities [50]. Some facilities do not offer MOUD at all, some allow only the continuation of MOUD and do not practice inductions, and some provide only certain types of MOUD [50]. At the state level, prisons offer continuation of buprenorphine and naltrexone [51]. Therefore, overall, MOUD access remains limited in correctional settings across the state but varies widely by locality. This study complies with the consolidated criteria for reporting qualitative research checklist (COREQ—see Additional file 1) [52].

Recruitment procedures and eligibility criteria differed for the unique groups of participants. A targeted sampling approach was employed to recruit female participants (*N* = 20) who had previously enrolled in an MOUD treatment program. This was achieved through social media promotions, referral chains, and the distribution of flyers to MOUD programs in Pennsylvania. Eligibility criteria for participation included (1) providing informed consent, (2) being at least 18 years old, (3) residing in PA, (4) having a record of incarceration, probation, or parole, and (5) having a history of MOUD program enrollment [47, 48].

The recruitment of SUD treatment professionals (*N* = 12) involved contacting a list of 103 Substance Abuse and Mental Health Services Administration (SAMHSA)-certified Opioid Treatment Programs (OTPs) in PA. To qualify for participation in the interview, the providers need to (1) give informed consent, (2) practice within Pennsylvania, and (3) engage in one or more of the subsequent activities: prescribing MOUD, providing care to individuals undergoing MOUD treatment, and/or offering complementary behavioral modification counseling as part of a MOUD treatment program [47, 48].

The process of recruiting criminal legal professionals (*N* = 10) involved a multi-pronged approach, including making direct contact with jails and prisons in PA, utilizing referrals, and advertising online. To qualify for participation, the professionals had to (1) consent to participate, (2) be involved with women who use or have used opioids, (3) be practicing within PA and (4) hold one of the specified roles: treatment court professional, parole or probation officer, or judge [47, 48].

To ascertain the fulfillment of eligibility requirements by all prospective participants, a brief web screening process was implemented. This involved the utilization of a REDCap survey, which gathered essential information such as age, gender, other demographic details, and contact information. The REDCap survey also confirmed participants’ eligibility for the study, which automatically concluded for potential participants who did not meet the predetermined criteria in the survey.

After recruitment, qualified participants who expressed interest were contacted by trained personnel from The Pennsylvania State University Survey Research Center to arrange their interview appointments. All participants agreed to be interviewed, and while no one refused, a few faced scheduling conflicts that prevented them from scheduling an appointment. The one-on-one interviews between the interviewer and participant lasted about an hour and were conducted over the telephone. During this study period, the interviewers were all employed as research interviewing staff, held PhDs or Masters in relevant social science fields, and had experience conducting research interviews. The interviewers, all of whom were women, shared demographic backgrounds similar to those of the women they interviewed who had experience with MOUD. No personal relationships were established before the interviews, and no personal details regarding the interviewers’ own lived experiences were disclosed to the participants.

Once the study details were clarified at the start of each call, participants verbally agreed to proceed, prompting the interview to commence upon their verbal consent. The semi-structured interview guide (see Additional file 2) explored participants’ own experiences with MOUD treatment or their experience working with women who received MOUD treatment. When discussing their experiences with various types of MOUD (methadone, buprenorphine, and/or naltrexone), the women referred to various treatment settings where they received MOUD, including correctional facilities, inpatient and outpatient treatment centers, OTPs, and other medical settings. They shared their experiences, both positive and challenging, using MOUD in these diverse settings. The interview staff emphasized that participants could decline to answer any questions they did not wish to answer. All participants received a $50 gift card as reimbursement for their time commitment.

Interviews were audio recorded, and these recordings were transcribed and securely stored within the Survey Research Center database. To assure participant confidentiality, transcripts were de-identified. The research team then conducted a thematic analysis, consistent with grounded theory [53, 54], using *NVivo 14* software. A team of three researchers first generated a list of primary codes inductively by carefully reading the transcripts. After these primary themes were generated, more specific secondary codes were created, again inductively, through repeated reading of transcripts to classify the fundamental categories and dynamics within each primary theme. This process of inductively generating codes indicated that the study had reached thematic saturation or redundancy in the interviews. Each transcript was then coded by one of three researchers, separate from those who generated the codes; to assure consistency, the principal investigator (PI) double-checked all coded transcripts, addressing and resolving any discrepancies through discussion with the primary coder [47, 48].

The present study draws on three secondary codes: experiences of detox, experiences with the criminal legal process, and reentry into society, all of which were under the ‘experiences related to opioid use’ primary code. In analyzing the text within these codes, we thematically organized the experiences reported by women. We then turned to the coded text within the professionals’ interviews to identify corroboration with the women’s accounts, any potential discrepancies (which there were none), and insight into the systemic reasons for women’s experiences.

## Results

In total, 42 individuals participated in the study. The affected women with a history of OUD and using MOUD (*N* = 20) had ages ranging from 24 to 54 years, with an average age of 37. Most identified as White and non-Hispanic, accounting for 70% of the group (*n* = 14). The sample also included 12 SUD treatment professionals, encompassing a diverse range of roles, including nurses (*n* = 2), counselors (*n* = 3), case managers/recovery coaches (*n* = 3), recovery/treatment program directors (*n* = 2), an MOUD provider (*n* = 1), and a research assistant (*n* = 1). Ten of the SUD treatment professionals were women, and 2 were men. Their ages ranged from 38 to 54 years, with an average age of 48. The majority of the SUD treatment professionals self-identified as White and not of Hispanic origin, comprising 66.7% of the group (*n* = 8). Finally, the sample included ten criminal legal professionals, including treatment court professionals (*n* = 4), law enforcement (*n* = 3), prosecutors (*n* = 2), and a corrections worker (*n* = 1). Seven of the criminal legal professionals identified as women, and three as men. One of the criminal legal professionals self-identified as Black, whereas nine individuals identified themselves as White and not of Hispanic origin. The average age of these participants was 44 years old, with ages spanning from 34 to 56 years [47, 48].

Through a thematic analysis, we found that women with OUD experience incarceration-related adversity in three distinct ways. Adversity evolved from (1) detox experiences at the beginning of their incarceration, (2) experiences of contempt from correctional staff and a lack of supportive resources during incarceration, and (3) experiences of hardship and stigma during reentry. Across these three domains, we also identified gender-specific hardships that intensified the adversity explicitly experienced by women with OUD.

The findings in this study shed light on the adverse effects of the detoxification process, particularly in local jails. The women we spoke to consistently expressed the severity of detox experiences while incarcerated in local jails, highlighting the challenging nature of this process. Additionally, professionals emphasized that the heightened potency of the substances used by women—particularly the growing use of fentanyl—has intensified the physical and psychological distress during the detox period.

One common theme was the lack of sufficient resources and support for individuals undergoing detox in jail. Participants reported a lack of detox programs or specialized interventions to alleviate the distressing symptoms of withdrawal. Several women noted that pregnant individuals were the only ones provided with any form of support during detoxification. One participant (#28) told us:

“The jails that I always got sent to, unless you were pregnant, they didn’t do anything for your detox. They would literally give you maybe something to keep [your] blood pressure down, but that was it. If you were bad enough, they’d give you a shot of Zofran [medication to reduce negative side effects including nausea] to keep you from throwing up so much. I remember being in jail and detoxing so badly that they were like, ‘Look you’re considered emaciated.’ I was so skinny and losing so much weight from throwing up and being so sick that they were making me drink these weird shakes in the morning and stuff.

I couldn’t keep anything down, and you go through the shakes. It’s so horrible. You’re throwing up, you have the runs... When they brought the food into the jail for lunch or breakfast, once you smell the food, you just start throwing it up and [start] gagging, it’s so bad. You get the restless leg syndrome where your legs are kicking all night, sweating, hot and cold. Oh my god, it sucks.”

This participant described in vivid detail how extreme it was to detox in jail, as she had done so not once but multiple times. Each time, she experienced weight loss, vomiting, diarrhea, and tremors, and despite this, she received little to no support to make the process any more comfortable.

A similar account was shared by another participant (#24), who also experienced extreme weight loss and constant vomiting during her detox period in jail. She explained:

“I sat there and pooped myself, and threw up, and chills, and fever and aches, and pains... That’s withdrawal. That’s worse than going through labor, honestly. That was awful. I looked awful. I was grey. I walked out there 40 pounds lighter in 14 days. I didn’t eat or drink or anything in there.”

This participant continued with a plea that detoxification should not have to take place in such a harsh and uncomfortable environment. She found that experiencing such symptoms without empathy or support was particularly dehumanizing beyond the dehumanization of incarceration alone.

Valuable perspectives on the detox experience were also contributed by some of the criminal legal professionals interviewed in the study. Their insights shed light on the challenges and limitations of the criminal legal system concerning detoxification processes. One drug court-certified recovery specialist (participant #11) noted:

“There’s no detox protocol [in jail]. The fentanyl is so prevalent right now, and the withdrawal from that is worse than anything I’ve ever seen. Seeing someone go through that while locked in an 8-by-10 cell with someone else is just devastating.”

This additional perspective provides insights into the nature of the detoxification experience, not just for the women who detox upon entering jail themselves but also for the women in jail with them watching their hardship. It further illuminates the profound challenges and adverse effects that women encounter during this critical period.

It is important to note that these experiences were specific to local jails rather than state and federal prisons. Most of the women in our sample had spent time in local jails for minor drug-related crimes and probation revocations; incarceration in state prisons was much less common. Some of the criminal legal professionals in our sample pointed out that there was greater support for the detox process in state prisons. Participant #13, a state prosecutor, stated:

“If they’re in prison, my understanding is that they do have help with withdrawal symptoms. To my knowledge, in all of the prisons, there are medical units that take care of withdrawal symptoms. I believe they stay in an intake unit before going into general population in the prison to make sure that they’re healthy and able to go into the general population.”

This participant suggested that individuals receive better support for withdrawal symptoms in state prisons through the provision of dedicated medical units. They asserted that it was common practice for individuals going through withdrawal to be properly cared for before joining the general prison population to ensure their health and readiness for integration.

In addition to describing detox experiences in jail, the women in this study unveiled accounts of experiencing both internal shame and various forms of abuse, contempt, and disregard within correctional settings, again, particularly in local jails. These included instances of verbal harassment, exploitation, and a lack of access to necessary healthcare and support services. One participant (#39) who was incarcerated in the same local jail as her son recounted:

“I wanted to jump out of a plane for my 50th birthday, and I ended up sitting in [county jail]. It’s horrible, especially if your son’s there and you’re there. It’s a bad feeling, man. It’s just terrible. Your mom’s here? It’s like, wow. I felt so bad for him. Everybody knows that your mom’s there. I feel like that’s so embarrassing for that kid. He’s been through a lot. He’s seen a lot.”

This participant revealed the shame that she experienced while incarcerated, not only because she lacked the freedom to celebrate her birthday but also because of the embarrassment she felt for her son. Being in the same jail as her son made her feel as if she failed him because of her own substance use.

Like participant #39 above, participant #24 also acknowledged feeling humiliated for being incarcerated. But she told us this humiliation did not come about internally but rather from the mistreatment she faced at the hands of correctional staff:

“Especially the women in [county jail] get treated very badly... There’s a point they basically say, ‘Okay, obviously, you’re not learning,’ but it’s a disease. They treat you like shit at these places [jail]... That was implanted in my head because drug addicts are a piece of shit. I was a piece of shit. The warden would look at us and say, ‘It’s not a women’s prison.’ He hated having women there. I watched horrific things go on there with the women... Especially if you come on into drugs too, they take advantage of you, it’s even worse.”

This participant described the contempt that she experienced from jail staff and explained how the stigma affected her self-perception thereafter. She also acknowledged that mistreatment in jails tends to be worse for women than men, especially for women who enter under the influence of drugs.

While participant #24 illustrated the unique gender dynamics by which women in jail were abused and dehumanized by primarily male staff, taking a toll on their mental health and self-image, other women described the additional challenges inflicted by incarceration when they had children on the outside. Participant #31 violated her probation by missing a required meeting with her probation officer because she was giving birth. She received a 35-day sentence and had to be separated from her newborn, whom she had been breastfeeding. Elaborating on the agony inflicted by this separation, she expressed:

“I had to get ripped away from my newborn son, even though I was clean, and be in jail and go through all that. It was the worst thing I’ve ever went through. It’s a point in my life, the last four years, where things really took a turn for the worst, I was just like, why does it matter if I’m clean or not? They’re still going to put me in jail. It doesn’t matter. It doesn’t matter if I try or don’t. Look at what they just did to me. My son had problems. I’m in jail bleeding from giving birth and leaking milk out of my boob. My son, every time I call home, he was screaming and crying from getting switched to formula overnight.”

This participant had her probation revoked for missing a probation meeting, even though she had not been using and had not failed a drug test. Being incarcerated, especially at such a fragile and vulnerable time in both her and her son’s lives, reminded her that it did not matter to the criminal legal system if she abstained from substance use or not; she could still wind up in jail. And if not using could not protect her freedom, then her motivation not to use decreased. Her experience suggests that experiencing penalties even when abstaining from substance use does not incentivize individuals to quit but, instead, may even heighten their motivation to continue using.

Yet further, participant #31’s story, especially, reminds us that we should question whether confinement is even necessary and useful for many women with OUD.

Notable disparities in resources and programs available to women in local jails compared to men were also revealed in the interviews. Participants highlighted that most jails’ resources, including MOUD access, substance use counseling, and mental health counseling, were primarily geared towards men due to their higher numbers, resulting in limited support for women. For instance, a treatment court coordinator (participant #17) mentioned the following:

Respondent: “It really depends on the county, but even in my old county..., there were many more programs available in the jail for men than there were for women. It almost feels like, for a woman, if they don’t go through a treatment court, they’re pretty limited on getting any help at all while they’re incarcerated. Whereas men, there’s so many different programs they can do.”

Interviewer: “What is the reason that there’s such an imbalance between resources for men versus women within the jail system? Is it a population difference?”

Respondent: “From the counties I’ve been in, it is really strictly a population difference because we would have, like, I’m trying to think how many thousand we had, 2000, 3000, maybe more prisoners incarcerated that were male, whereas females, it may be just several hundred... They didn’t have enough population to start a very involved program, whereas, on the male side, they did.”

These findings underscore the disparities that women face with limited access to MOUD treatment unless they go through a treatment court. Moreover, according to the account of this participant, the lack of resources available to women results from them representing a much smaller portion of the incarcerated population.

While incarceration itself can be challenging, the period following release is often marked by significant struggle and difficulties as well. Individuals reentering society face many challenges that can impede their successful transition. They may encounter difficulties securing stable housing, finding employment opportunities, and rebuilding relationships with family and friends. The stigma associated with incarceration can also lead to social isolation and limited support networks, exacerbating feelings of loneliness and alienation.

Moreover, participants told us that these challenges were heightened for women compared to men. One treatment court coordinator (participant #17) stated:

“The females are more isolated when they get out of incarceration, and they don’t know any resources, they don’t know where they can get housing, what they can do. They go back to what they know. That’s just going to keep being a pattern because they didn’t get the help they need.”

This person highlights that they felt women were more likely to relapse because they lacked the support and resources that men were more likely to have.

The experiences of the women we interviewed corroborated participant #17’s point. Participant #31, for example, illustrated an instance where her post-incarceration medical needs were unmet, highlighting the lack of necessary assistance provided in the reentry process:

“I knew I was going to relapse. I couldn’t get the meds back. They wouldn’t give them to me. I know how much better I was doing with the right meds. Now I don’t have them, and I’m struggling. I’m going to end up using. She [the psychiatrist she started seeing after release] wouldn’t give me the meds back.”

This participant has underlying mental health issues that trigger her substance use. She wanted to continue her mental health medication to help her avoid using drugs; however, she was unable to find a medical provider that would prescribe it to her upon her release. This led her to feel as if the providers held a stigma towards her because of her criminal legal involvement and SUD. Since she was unable to access the medications she needed, she worried about being at risk of relapsing.

Apart from the inadequate availability of resources and medical assistance for women after their release from incarceration, the additional burden of societal stigma attached to their criminal history further exacerbates the challenges they face. While participant #31 discussed how stigma impacted her interactions with medical professionals, many others told us how this stigma impacts job opportunities. Further, they suggested that stigma’s impact on job opportunities affects women more significantly than men. As a result, women experience not only face financial hardships but also psychological distress stemming from limited employment opportunities and associated societal judgment. When asked if being incarcerated had a long-term impact on her life, participant #29 responded:

“Oh, greatly. Anytime I would look for a job after that, you’re reminded that you’re a felon and a criminal because you do not get hired. You don’t. It is so hard.

It affects my financial situation. I then also feel depressed because I’m not contributing to my household, and I can’t just have that ability to help out or go take the kids shopping when I want to. It just plays on your self-esteem in so many ways.”

This participant described how her inability to find employment takes a toll on her self-esteem because she cannot provide for her family financially, leading her to use it to make herself feel better.

Given the complexities that come with reentry, stress, and hardship can trigger individuals with OUD to use drugs. Participant #35, for example, shed light on the numerous challenges and temptations that arise during reentry to society, making it incredibly easy to slip back into familiar patterns:

“You’re excited about getting out, and then I go back to the same neighborhood, the same apartment. Then you start seeing the same people and it’s just too much to handle... You put somebody that has my illness, sitting there being bored and stressing about all kinds of things you have to do and didn’t do and kids and stuff. You get out and reality sets in, and you just want to numb your pain.”

This participant underscored the feeling of anticipation for release, only to return to an unsupportive environment, influencing them to use it yet again.

The lack of comprehensive reentry programs and limited resources further compounds the hardships faced by women with OUD who are reentering society after incarceration. The process of rebuilding their lives to successful reintegration often meets inevitable adversity, potentially leading to relapse for women who seek to abstain from illicit substances post-incarceration.

## Discussion

This study aimed to identify challenges associated with involvement in the criminal legal system among women with OUD. We interviewed women with OUD, SUD treatment professionals, and criminal legal professionals to gain a more comprehensive understanding of how the adverse experience of criminal legal involvement impacts the substance use trajectories of women with OUD. We found that women experienced adversity in three significant ways: undergoing the detox process upon entering local jails, experiencing mistreatment and lack of support during incarceration, and facing challenges associated with reentry post-incarceration.

While previous studies have highlighted the pervasiveness of OUD among women in the criminal legal system [23, 24] and the lack of access to MOUD for incarcerated women with OUD [25, 27, 28], to our knowledge, the current study is the first of its kind to examine how the detoxification process serves as a challenging experience for incarcerated women with OUD. Many of the women detailed their experiences of detox while in jail, emphasizing the urgent need for improved support and resources to address the unique needs and vulnerabilities of women undergoing detoxification in jail. The absence of appropriate medical care left the women to endure severe withdrawal symptoms. Corroborating these firsthand accounts, insights from SUD treatment professionals emphasized that the heightened potency of the substances used by women, notably the increasing prevalence of fentanyl, has intensified both the physical and psychological distress during the detox period. This claim is supported by current literature suggesting that fentanyl has significantly contributed to the rise in opioid overdoses [55] and has an estimated potency ranging from 30 to 50 times that of heroin and 50 to 100 times of morphine [56].

Moreover, SUD treatment and criminal legal professionals concurred with the recollections of the women with OUD, providing additional support for the need to improve how the detox process is managed within correctional settings, particularly within local jails. These findings corroborate existing studies that show that local jails have significantly fewer resources than state and federal prisons and often fail to provide adequate care during detox [57, 58]. They highlight a critical need to examine detox and treatment resources across localities and ensure adequate medical care is offered to incarcerated people regardless of their place of residence.

The women in our study also often mentioned experiencing internal shame, contempt from corrections officers, and a lack of supportive resources while incarcerated. While many women felt personal shame for their circumstances, these feelings were often heightened through experiences of abuse and belittlement by correctional staff. These findings highlight the need for systemic reforms and targeted interventions to address the mistreatment of women in the criminal legal system, ensuring their safety, dignity, and rights are upheld during their time of confinement. Previous studies have focused on physical and sexual victimization during incarceration [34, 35] but our study adds to the literature by revealing the effects of verbal abuse and insolence.

Moreover, several of the participants in our study also spoke to a lack of treatment and services for incarcerated women with OUD, while men had more opportunities to participate in programs related to substance use during their time incarcerated. Again, this lack of support and resources for women is heightened in local jails, where women are fewer in number and resources in general are less robust. These findings warrant more equitable approaches within the legal system to ensure that all individuals, regardless of their gender, receive the necessary support and resources to address addiction and related issues during this time. The stigma associated with OUD in the criminal legal system is a well-known barrier to accessing proper resources and support [59–61]. Although only a few correctional institutions offer MOUD treatment while incarcerated [61], several studies have found benefits to treatment utilization during incarceration, including the decreased likelihood of illicit drug use, decreased likelihood of overdose, and increased probability of treatment continuation post-release [62–64].

Unfortunately, the challenges encountered by the women in our study while reintegrating into society, which stemmed from the stigmatization of OUD and the absence of adequate resources, contributed to their substance use. The women experienced challenges accessing MOUD and mental health medications when they left jail, contributing to the likelihood of relapse. Others were faced with difficulty securing employment due to the stigma of their criminal history, which could impose significant stressors that lead to motivation to use. Furthermore, participants spoke about the mental distress they experienced when they could not provide for their families, a challenge that has been commonly observed in previous research [65–67]. Although post-incarceration adaptation research is limited, the transition back into society can be a stressful life event due to the disruption in daily routine [65]. Our findings suggest that this transition can be even more difficult for women also navigating OUD and early recovery. For women with OUD, successful reentry demands a multi-faceted approach, encompassing systemic changes along with both individual and community support. This approach can provide individuals with the opportunities and support they need to reintegrate successfully, avoid relapse, and thrive in society post-incarceration.

While this study significantly enriches the current literature, a few limitations exist. First, given the cross-sectional nature of the study design, the study results depended solely on retrospective accounts, which may introduce the potential for inaccuracies. Also, the results may have been subject to respondent bias, as participants could have been inclined to provide more admirable answers in the interviews. However, since interviews were conducted over the phone rather than in person, this may alleviate this concern. Furthermore, because our study only included women with OUD, who often spoke to the additional difficulties faced by women as compared to men, we could not directly compare the experiences of men and women with OUD.

Some limitations identified in our study offer valuable insights for future research. Our study recruited participants exclusively from one state in the northeast, potentially limiting the generalizability of our findings to other regions. Additionally, the majority of women with OUD in our study (70%) self-identified as White, and their ages ranged from 24 to 54 years old, which may not accurately represent the population of women with OUD. Furthermore, due to the reliance on self-report data and semi-structured interviews, not all participants were able to provide consistent information about contextual factors related to substance and MOUD use, including type and timing. Yet, obtaining such information would have enriched their narratives, providing further depth to their experiences. For instance, it could have shed light on whether their substance use experiences coincided with the fentanyl crisis [68, 69] or predated the availability of MOUD in carceral settings [70, 71]. Moreover, a detailed analysis of participants’ incarceration histories was not feasible as not all participants disclosed specific information about their incarceration locations, timing, or duration. Consequently, we were unable to consider the influence of rural versus urban settings on participants’ experiences within the criminal legal system in our study. Future research should account for geographic variations in criminal legal experiences and gather precise data on MOUD usage, as MOUD experiences are diverse and may significantly influence women’s substance use trajectories.

## Conclusions

In conclusion, we find that involvement in the criminal legal system has considerable adverse impacts on women with OUD. The reality of the detox process and adverse conditions within correctional settings, particularly local jails, emphasize the necessity for more universal and comprehensive detox protocols. Forthcoming investigations should also assess, and address adversity experienced during incarceration, as well as challenges associated with reintegration into society. The findings from this study suggest further exploration of trauma-informed interventions aimed to increase support for women with OUD while incarcerated and facilitate their adaptation to life post-release.

## Supplementary Information

**Additional file 1: Table S1.** COREQ (COnsolidated criteria for REporting Qualitative research) Checklist. The COREQ (Consolidated Criteria for Reporting Qualitative Research) checklist is a tool that helps ensure transparent and thorough reporting of qualitative research studies, which includes 32 items covering aspects such as research team details, study design, and analysis methods.

**Additional file 2.** Semi-Structured Interview Guide. The interview guide is designed as semi-structured, facilitating participants to provide comprehensive insights into their experiences concerning drug use, drug treatment, and interactions with the judicial system. Interviewers are directed to actively listen for pertinent themes in these areas and employ probing techniques as necessary during the interview process.

## Data Availability

Due to the sensitive nature of the topics discussed in the interviews, these data are not publicly available. Data may be made available upon reasonable request by contacting the P.I. (Abenaa Jones, avj5462@psu.edu).

## Abbreviations

SUD: Substance use disorder
MOUD: Medication for opioid use disorder
OUD: Opioid use disorder
DSM V: Diagnostic and Statistical Manual of Mental Disorders, 5th Edition
DSM IV: Diagnostic and Statistical Manual of Mental Disorders, 4th Edition
NIS: National Inmate Survey
PTE: Potentially traumatic event
PTSD: Post-traumatic stress disorder
PA: Pennsylvania
SAMHSA: Substance Abuse and Mental Health Services Administration
OTP: Opioid Treatment Programs
PI: Principal investigator
